# Treating Type 2 Diabetes With Early, Intensive, Multimodal Pharmacotherapy: Real-World Evidence From an International Collaborative Database

**DOI:** 10.1155/2024/3470654

**Published:** 2024-05-30

**Authors:** Matthew Anson, Ayesha Malik, Sizheng S. Zhao, Philip Austin, Gema H. Ibarburu, Shabbar Jaffar, Anupam Garrib, Daniel J. Cuthbertson, Uazman Alam

**Affiliations:** ^1^ Diabetes & Endocrinology Research and Pain Research Institute Institute of Life Course and Medical Sciences University of Liverpool and Liverpool University Hospital NHS Foundation Trust, LiverpoolUK; ^2^ School of Medicine Barts and the London Queen Mary University of London, LondonUK; ^3^ Centre for Musculoskeletal Research Division of Musculoskeletal and Dermatological Science School of Biological Sciences Faculty of Biological Medicine and Health The University of Manchester Manchester Academic Health Science Centre, ManchesterUK; ^4^ TriNetX LLC, Cambridge, MassachusettsUSA; ^5^ UCL Institute for Global Health University College London, LondonUK; ^6^ Diabetes & Endocrinology Research Institute of Life Course and Medical Sciences University of Liverpool and Liverpool University Hospital NHS Foundation Trust, LiverpoolUK; ^7^ Visiting Fellow Centre for Biomechanics and Rehabilitation Technologies Staffordshire University, Stoke-on-TrentUK

**Keywords:** cardiovascular outcomes, ominous octet, pioglitazone, type 2 diabetes

## Abstract

**Aims:** We compared the glycaemic and cardiorenal effects of combination therapy involving metformin, pioglitazone, sodium-glucose-linked-cotransporter-2 inhibitor (SGLT2i), and glucagon-like peptide-1 receptor agonist (GLP-1RA) versus a more conventional glucocentric treatment approach combining sulphonylureas (SU) and insulin from the point of type 2 diabetes (T2D) diagnosis.

**Methods:** We performed a retrospective cohort study using the Global Collaborative Network in TriNetX. We included individuals prescribed metformin, pioglitazone, an SGLT2i, and a GLP-1 RA for at least 1-year duration, within 3 years of a T2D diagnosis, and compared with individuals prescribed insulin and a SU within the same temporal pattern. Individuals were followed up for 3 years.

**Results:** We propensity score-matched (PSM) for 26 variables. A total of 1762 individuals were included in the final analysis (*n* = 881 per cohort). At 3-years, compared to the insulin/SU group, the metformin/pioglitazone/SGLT2i/GLP-1 RA group had a lower risk of heart failure (HR 0.34, 95% CI 0.13–0.87, *p* = 0.018), acute coronary syndrome (HR 0.29, 95% CI 0.12–0.67, *p* = 0.002), stroke (HR 0.17, 95% CI 0.06–0.49, *p* < 0.001), chronic kidney disease (HR 0.50, 95% CI 0.25–0.99, *p* = 0.042), and hospitalisation (HR 0.59, 95% CI 0.46–0.77, *p* < 0.001).

**Conclusions:** In this real-world study, early, intensive polytherapy, targeting the distinct pathophysiological defects in T2D, is associated with significantly more favourable cardiorenal outcomes, compared to insulin and SU therapy.

## 1. Introduction

Type 2 diabetes (T2D) is a substantial public health issue. It is estimated that by 2030, ~20% of individuals globally will be affected by dysglycaemia [1]. The worldwide population living with T2D will be 578 million, with a further 454 million people projected to live with impaired glucose tolerance [1]. T2D is associated with significant cardiorenal disease, and the complications of diabetes account for substantial costs, with the management of macrovascular disease being a significant economic burden, exceeding that of microvascular disease [2].

The pathophysiology of T2D is a complex interaction between an at-risk genotype, ethnicity, diet, lifestyle, and socioeconomic factors all interacting and contributing to dysglycaemia. Pathophysiological defects include whole-body insulin resistance characterised by increased fasting hepatic glucose production, reduced suppression of hepatic glycogenolysis and gluconeogenesis by insulin, and reduced (postprandial) skeletal muscle and adipose tissue glucose uptake [3]. Pancreatic *β* cells produce a compensatory increase in insulin secretion, and thus euglycaemia is initially maintained; however, there is progressive failure of *β* cell function.

This traditional triad of *β* cell failure, muscle, and liver insulin resistance was expanded by introducing five additional factors, the “ominous octet” [3], that contribute to the pathophysiology of T2D, including (1) increased peripheral lipolysis and NEFA mobilisation, (2) reduced secretion of and increased resistance to glucagon-like peptide-1 (GLP-1) and glucose-dependent insulinotropic polypeptide (GIP), (3) hyperglucagonaemia from a reduced incretin response and *α* cell insulin resistance, (4) increased renal glucose reabsorption threshold, and (5) appetite dysregulation.

The current management of T2D is conducted in a graded and stepwise manner, with therapeutic inertia recognised as a significant barrier to achieving optimal clinical outcomes [4]. The prioritisation of glucose lowering in T2D, at the expense of remedying the “ominous octet” is reflected in prescribing data [5]. The median time for an additional antidiabetic agent to be added to initial therapy is 2 years, with further intensification taking on average 7 years [6], with clinical decision-making often reactionary rather than proactive.

GLP-1 receptor agonists (GLP-1 RAs) offer extraglycaemic benefits (reduced appetite, weight loss, and a protective effect on pancreatic islets [7]), but are only offered in some regions when multiple oral agents have failed to achieve target HbA1c, subject to weight criteria [8]. Sodium-glucose-linked-cotransporter-2 inhibitors (SGLT2is) offer cardiorenal protection, but early adoption is only encouraged with the presence of concomitant atherosclerotic cardiovascular disease (ASCVD) or chronic kidney disease (CKD) [9]. Thiazolidinedione therapy has been more limited in recent times [10]. The risks associated with fluid retention, fractures, and the removal of rosiglitazone from the market have restricted their widespread use, despite their significant insulin sensitising properties and positive cardiovascular outcome data, even in the absence of diabetes [11].

Insulin and insulin secretagogues are the most potent glucose-lowering therapies available in T2D [12]. However, such treatments remedy only one of the metabolic derangements of the ominous octet, namely, reduced insulin secretion. Sulphonylureas (SU) augment insulin secretion from an already diminishing pool of functioning *β* cells and can further accelerate their decline [13]. Exogenously administered insulin offers a different and suboptimal metabolic profile compared to endogenous secretion. Secretagogues and exogenous insulin are metabolically inferior to endogenous insulin and fail to correct the complex underlying pathophysiology.

Treating the various pathophysiological defects in T2D early, immediately after diagnosis, is rarely undertaken, with the effects on long-term cardiovascular outcomes unknown. Therefore, we aimed to investigate the long-term real-world effects of combination therapy with metformin, pioglitazone, GLP-1 RA, and an SGLT2i compared to an insulinocentric model of treatment with SU and insulin only, from the initial diagnosis of T2D.

## 2. Methods

### 2.1. Database

We performed a retrospective cohort study using the TriNetX (TriNetX LLC, Cambridge, MA, USA) platform. The TriNetX research platform is a global collaborative network providing access to real-time, anonymised electronic medical records. TriNetX has data usage and publication agreements in place with all healthcare organisations (HCOs). The TriNetX Global Collaborative Network composes of approximately 125 million patients across over 100 HCOs, primarily secondary and tertiary units in North America and Europe. Data contained in the network includes demographics, diagnosis (International Classification of Diseases, 10^th^ Revision (ICD-10)), procedures (Current Procedural Terminology (CPT) or ICD-10 Procedure Coding System), medications (drug classes coded in Veterans Affairs National Formulary (VA) or Anatomical Therapeutic Chemical classification (ATC), specific drugs coded in RxNorm terminology), laboratory values (Logical Observation Identifiers Names and Codes (LOINCs)), and healthcare utilisation. The database can be analysed in its entirety (global) or be limited to US or European networks. For our study, we utilised the global collaborative network. The data used in this study was collated on 8 August 2023.

### 2.2. Building Our Cohort

We included all individuals aged 18 or over with a diagnosis of T2D (ICD-10:E11). We excluded individuals who ever prescribed a dipeptidyl peptidase 4 inhibitor (DPP4i), alpha-glucosidase inhibitor, tirzepatide, and pramlintide. We created two cohorts of individuals termed (1) quadruple therapy: metformin (MTF)/thiazolidinedione (TZD)/GLP-1 RA/SGLT2i and (2) traditional/conventional therapy: insulin + sulphonylurea. For the quadruple therapy cohort, we included all individuals who were prescribed metformin, pioglitazone, GLP-1 RA, and SGLT2i within 3 years of diagnosis of T2D, and therapy must have been continued for at least 1 year. We excluded individuals who were prescribed insulin and SU within 3 years of diagnosis. For the traditional therapy cohort, we included all individuals who were prescribed insulin and a sulphonylurea within 3 years of diagnosis of T2D, and therapy must have continued for at least 1 year. In the latter cohort, we excluded individuals who were prescribed metformin, pioglitazone, any GLP-1 RA, or any SGLT2i within 3 years of diagnosis. The index event was defined as individuals on their respective treatment for at least 1 year.

### 2.3. Propensity Score Matching

Propensity score matching using a 1:1 ratio using greedy nearest neighbour matching with a caliper of 0.1 pooled standard deviations was utilised. We matched cohorts for baseline major cardiovascular risk factors and other common variables commonly associated with either a net positive or negative cardiovascular effect. We matched for age at index event, sex, race, essential hypertension (ICD-10:I10), atrial fibrillation and flutter (ICD-10:I48), heart failure (ICD-10:I50), ischemic heart disease (ICD-10:I20-I25), CKD (ICD-10:N18), antilipemic agents, ACE inhibitors, angiotensin II inhibitors, diuretics, beta-blockers, antiarrhythmics, platelet aggregation inhibitors, anticoagulants, low-density-lipoprotein cholesterol, HbA1c, body mass index, blood pressure, estimated glomerular filtration rate, alcohol dependence, nicotine dependence, problems related to housing and economic circumstances, and problems related to education and literacy. A strictly standardised mean difference (SSMD) of < 0.1 was considered to be well-matched.

### 2.4. Outcomes of Interest

Analysis of outcomes began 1 day after the index event up until the 3-year postindex event. Clinical outcomes recorded were death, chronic ischaemic heart disease (IHD) (ICD-10:I25), heart failure (ICD-10:I50), acute coronary syndrome (ICD-10:I21-I22), CKD (ICD-10:N18), end-stage renal failure (ESRF) (ICD-10:N18.6), stroke (ICD-10:I61,I63), and hospitalisation. Side effects recorded were hyperosmolar hyperglycaemic state (HHS) (ICD-10:E11), diabetic ketoacidosis (DKA) (ICD-10:E11.1), acute pancreatitis (ICD-10:K85), urinary tract infection (UTI) (ICD-10:N39.0), candidiasis (ICD-10:B37.3), osteoporosis (ICD-10:M80-81), and hypoglycaemia (ICD-10:E11.64). Laboratory and anthropometric values included HbA1c, eGFR, and body weight. The most recent value in individuals' electronic medical records for analysis was recorded. We excluded all individuals with a documented history of a clinical outcome of interest. However, individuals included who had a prior history of hospitalisation and adverse side effects of interest (with a notable exception of osteoporosis) as side effects are not typically associated with long-term, lasting sequelae compared to a new diagnosis of a chronic condition.

### 2.5. Statistical Analysis

All statistical tests are performed in situ within the TriNetX analytics platform. Hazard ratios (HRs) and associated confidence intervals are calculated using “R's survival package,” with the proportional hazard assumption tested using the generalised Schoenfeld approach. Statistical significance is set at *p* < 0.05.

## 3. Results

### 3.1. Baseline Characteristics Including Treatments

We identified 7,871,583 individuals with T2D within the network. All individuals who were ever issued a DPP4i, alpha-glucosidase inhibitor, pramlintide, or tirzepatide were excluded. A total of 35,891 individuals were identified for our study prior to PSM (quadruple and traditional therapy). Within 3 years of diagnosis of T2D, 882 individuals were treated with a combination of metformin, pioglitazone, SGLT2i, and GLP-1 RA for at least 1 year and without being prescribed any insulin or sulphonylurea for 3 years. Additionally, 35,009 individuals were treated with a combination of insulin and a sulphonylurea for at least 1 year, within 3 years of diagnosis of T2D, and without being issued metformin, pioglitazone, or any SGLT2i or GLP-1 RA within this time frame.

Individuals initiated on insulin and sulphonylurea were on average older and had a lower eGFR and greater HbA1c than those starting on quadruple therapy.  Table 1 summarises the whole cohort's baseline demographics and characteristics.

We identified 26 key variables (demographics, comorbidities, medication, and laboratory values) for PSM, of which 25 out of the 26 were well matched. The only variable that did not match well was “problems related to housing and economic circumstances,” where there was a greater proportion of individuals affected in the quadruple therapy cohort. After matching, individuals in the “MTF/TZD/GLP-1 RA/SGLT2i” group were reduced by 1 to 881 and individuals in the “insulin + sulphonylurea” group were reduced by 34,128 to 881.  Table 2 summarises the variables that we matched for.

### 3.2. Clinical Outcomes

**Figure 1:**
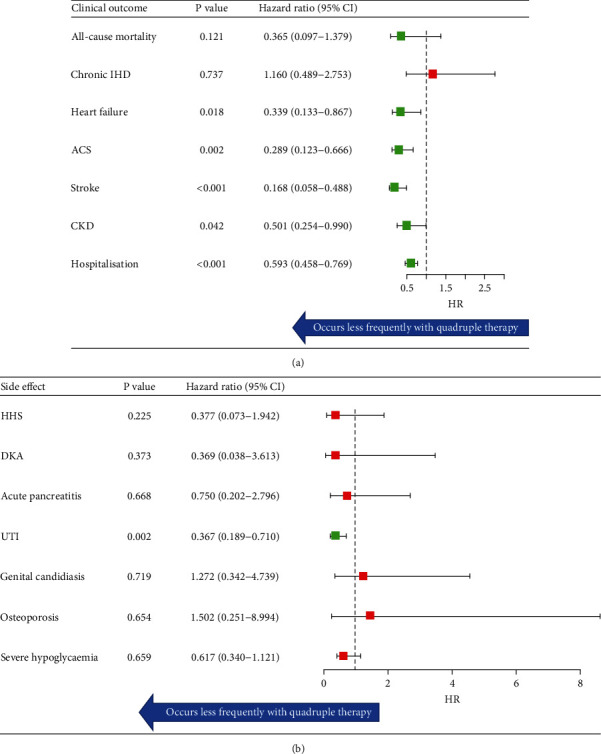
(a) Clinical outcomes over 3 years as a forest plot. Green boxes represent statistically significant findings. (b) Adverse clinical outcomes over 3 years as a forest plot. Green boxes represent statistically significant findings. IHD: ischaemic heart disease; ACS: acute coronary syndrome; CKD: chronic kidney disease; HHS: hyperosmolar hyperglycaemic state; DKA: diabetic ketoacidosis; UTI: urinary tract infection.

Individuals on quadruple therapy had a reduced risk of heart failure (HR 0.34, 95% CI 0.13–0.87, *p* = 0.018), ACS (HR 0.29, 95% CI 0.12–0.67, *p* = 0.002), stroke (HR 0.17, 95% CI 0.06–0.49, *p* < 0.001), CKD (HR 0.50, 95% CI 0.25–0.99, *p* = 0.042), hospitalisation (HR 0.59, 95% CI 0.46–0.77, *p* < 0.001), and UTI (HR 0.37, 95% CI 0.19–0.71, *p* = 0.002) compared to insulin + sulphonylurea therapy (Figure 1(a)).

### 3.3. Adverse Clinical Outcomes

There were no differences between all-cause mortality, development of chronic IHD, HHS, DKA, acute pancreatitis, genital candidiasis, osteoporosis, or significant hypoglycaemia between groups (Figure 1(b)). Ten individuals developed ESRF, and 10 individuals developed bladder cancer within the insulin + sulphonylurea group. No one within the quadruple therapy group developed ESRF or bladder cancer (*p* < 0.001).

### 3.4. Changes in Biochemistry

**Figure 2:**
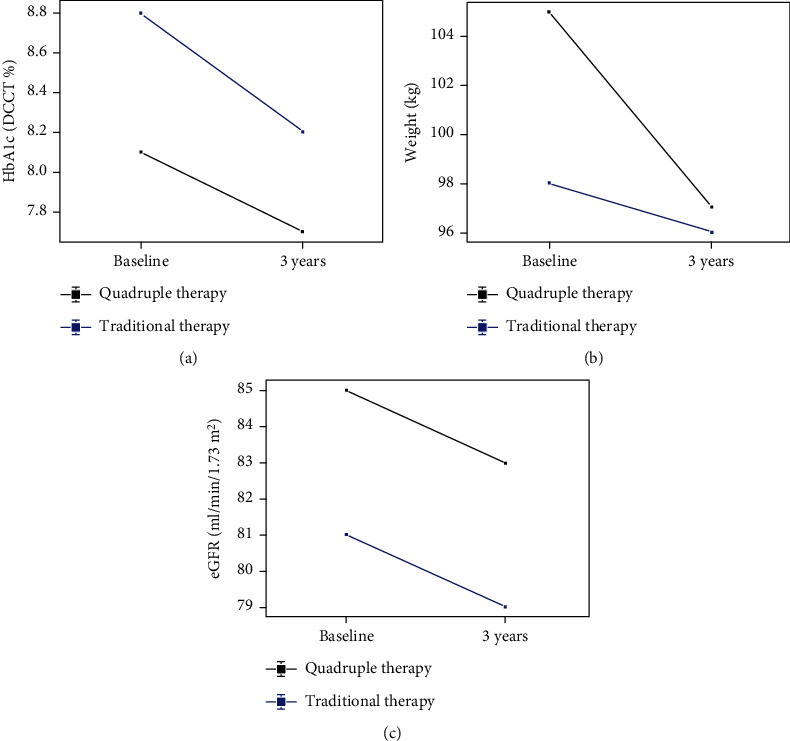
(a) Change in HbA1c over 3 years. Blue line represents the “insulin + sulphonylurea” group and black line represents the “MTF/TZD/GLP-1 RA/SGLT2i” group. (b) Change in weight over 3 years. Blue line represents the “insulin + sulphonylurea” group and black line represents the “MTF/TZD/GLP-1 RA/SGLT2i” group. (c) Change in eGFR over 3 years. Blue line represents the “insulin + sulphonylurea” group and black line represents the “MTF/TZD/GLP-1 RA/SGLT2i” group.

**Figure 3:**
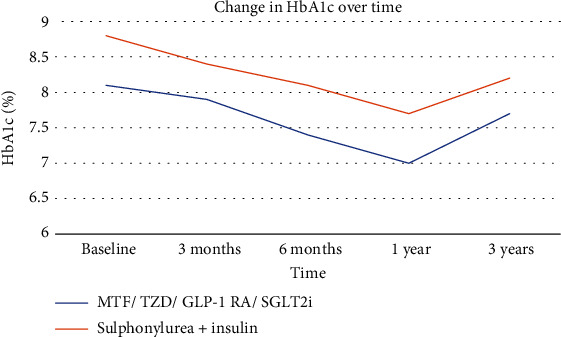
Change in HbA1c over time.

Individuals taking insulin + sulphonylurea had a higher starting HbA1c 8.8% ± 2.1% (73 mmol/mol ± 23 mmol/mol) versus 8.1% ± 1.5% (65 mmol/mol ± 17 mmol/mol) and had a greater reduction in HbA1c at 3 years −0.6% (−7 mmol/mol) versus −0.4% (−4 mmol/mol) than individuals initiated on quadruple therapy (Figure 2(a)). Maximal HbA1c reduction was seen at 1 year in both groups −1.1% (−12 mmol/mol) and was comparable between both arms (Figure 3). Both groups had similar minor reductions in eGFR over 3 years (Figure 2(c)).  Table 3 summarises these findings at 3 years.

### 3.5. Changes in Body Weight

Individuals starting on quadruple therapy had a higher baseline weight but demonstrated substantially more weight loss (−7.6 kg vs. −1.2 kg) compared to the insulin + sulphonylurea group (Figure 2(b)).

## 4. Discussion

We have undertaken the largest real-world study to date adopting a treatment approach, with a broad range of glucose-lowering agents, that specifically targets the pathophysiological defects of the “ominous octet'” versus primarily glucocentric agents. Our study demonstrates that “targeted” combination therapy (with metformin, pioglitazone, SGLT2i, and GLP-1 RA), early after diagnosis of T2D, confers superior cardiorenal outcomes when compared to older, conventional glucose-lowering agents that have no other specific mechanism of actions. Such individuals, treated with this targeted pharmacological approach, had a significantly reduced risk of heart failure, ACS, stroke, CKD, and hospitalisation for any cause despite similar glucose-lowering efficacies. Both groups had similar magnitudes of reduction in HbA1c at 1 and 3 years and comparable declines in eGFR between treatment regimes. Individuals treated with quadruple therapy had greater weight loss (−7.6 kg vs. −1.2 kg) than those treated with SU + insulin only. It is not surprising that increased weight loss was observed in individuals treated with GLP-1 RA and SGLT2i, but it was encouraging to see that weight loss was maintained even in the presence of a thiazolidinedione, an agent which is associated with fluid retention and weight gain. This is in keeping with our previously published meta-analysis where a combination of pioglitazone with GLP-1 RA or SGLT2i was associated with a greater degree of weight loss compared to monotherapy [14]. Even after an absolute minimum period of 1 year on our cohorts' respective therapy, we observed reductions in incident adverse cardiovascular outcomes at 3-years with the metformin/TZD/GLP-1/SGLT2i arm. Quadruple therapy reduced the risk of incident heart failure by 66%, ACS by 71%, stroke by 83%, CKD by half, and hospitalisation for any cause by 40% over a 3-year period.

The Efficacy and Durability of Initial Combination Therapy (EDICT) for T2D trial offers proof of concept of the validity of the ominous octet model [15]. EDICT is the only study to randomise individuals to receive either triple therapy with metformin, pioglitazone, and exenatide versus an escalating dose of metformin with the sequential addition of sulphonylurea and insulin glargine. The authors reported that triple therapy improved insulin sensitivity and *β* cell function and offered a greater and longer-lasting reduction in HbA1c than conventional therapy. A 3-year follow-up of EDICT highlighted a threefold increase in insulin sensitivity, a thirtyfold increase in *β* cell function, and durability of HbA1c reduction in the triple therapy arm compared to the conventional group, despite the escalation of treatment in the latter [16]. SGLT2i were not established in the management of T2D during the study period and as such were not included. The vildagliptin efficacy in combination with metformin for early treatment of T2D mellitus (VERIFY) trial additionally found a benefit of initial dual therapy compared to metformin monotherapy, with durable effects on HbA1c persisting for at least 3 years [17]. A Taiwanese study reported an over 30% risk reduction in new heart failure with a combination therapy of metformin and an SGLT2i [18]. Despite encouraging data, initial combination treatment for T2D still remains the exception to routine practice.

Suboptimal glycaemic control in the early years of diagnosis is associated with worse clinical outcomes, increased mortality, and incidence of diabetes-associated microvascular disease: a phenomenon termed the “glycaemic legacy” [19]. Immediate and intensive therapy may therefore be required to mitigate these risks. The management of diabetes attaches importance on the normalisation of HbA1c, considering irrefutable evidence between elevated HbA1c and the development of diabetic micro- and macrovascular complications [20]. Indeed, international guidelines do not stipulate that the selection of pharmacotherapy initiation should be based on addressing multiple aberrant metabolic pathways in T2D. However, our findings suggest merit in this more targeted approach with the demonstration that this may be associated with beneficial cardiorenal outcomes compared to glucocentric targets. Importantly, our study demonstrated benefit in a cohort without preexisting cardiorenal disease. Our previous real-world data study has demonstrated that combination therapy with SGLT2i and GLP-1 RA conferred mortality and cardiovascular protection in T2D over 5 years, particularly in all-cause mortality [21]. Current joint ADA (American Diabetes Association) and EASD (European Association for the Study of Diabetes) guidelines [9] suggest initiating GLP-1 RA or SGLT2i instead of metformin as first-line therapy only in individuals with high ASCVD risk factors. In individuals with low ASCVD risk factors, metformin remains as first-line therapy. GLP-1 RA currently only features as third- or fourth-line therapy in the UK's National Institute for Health and Care Excellence (NICE) [8] and in select individuals only, with strict criteria to continue therapy.

Both GLP-1 RA and thiazolidinedione preserve *β* cell function in humans [22], while in mouse models, SGLT2i have a protective effect on *β* cells, independent of their glucose-lowering effect [23]. Therefore, there is a good rationale for the early adoption of therapies that promote *β* cell function and have cardiovascular benefits. Data from a large Italian registry reported that patients newly diagnosed with T2D with poor initial glycaemic control were at higher risk of cardiovascular disease, but this risk is mitigated when SGLT2i are used within 2 years of diagnosis [24]. Recently, semaglutide treatment in early type 1 diabetes (T1D) has shown a reduced requirement of prandial insulin suggesting a beneficial impact on *β* cell preservation [25]. Our recent study also demonstrated that SGLT2i and GLP-1 RAs have potential benefits as adjunctive agents in T1D, with SGLT2i providing the greatest cardiorenal benefits [26].

The PROactive trial [27] reported a significant reduction in all-cause mortality, nonfatal myocardial infarction, and stroke with pioglitazone. The Insulin Resistance Intervention after Stroke (IRIS) study [28] reported a 25% reduction in a composite of stroke or myocardial infarction with pioglitazone poststroke or TIA, in individuals without diabetes but with insulin resistance. The progression of insulin resistance to frank diabetes was reduced by more than half. These cardiovascular benefits are consistently translated into larger real-world studies which support significant reductions in major adverse cardiovascular outcomes with pioglitazone use [29–31].

This study has limitations inherent to real-world data. We could not stratify by type of GLP-1 RA, SGLT2i, or insulin used, nor do we have access to dosing regimens. Additionally, the order of treatment escalation is unknown, and beyond the 1 year of treatment compliance stipulated in our methodology, the effect of any subsequent changes in the medication regime is also unknown. However, despite these limitations, we demonstrate that intensive treatment has beneficial cardiorenal outcomes at 3 years. Individuals in the “insulin and sulphonylurea” arm had a higher baseline HbA1c on average which may influence outcomes, but the proportion of individuals with a HbA1c ≥ 7.5% was well balanced. We defined relatively strict inclusion criteria, with the intensification of treatment occurring within 3 years of diagnosis. In typical clinical practice, time to reach combination therapy typically takes more than double this length of time. The intention behind a rapid treatment intensification was to demonstrate the impact of minimising treatment inertia and subsequent poor glycaemic legacy deleteriously influencing later outcomes. We relied upon ICD coding of hypoglycaemia. Most episodes of hypoglycaemia do not require formal medical attention/hospitalisation and hence, we can only report on clinically significant and severe hypoglycaemia. Although there was a trend for less hypoglycaemia with quadruple therapy, the upper boundary of the confidence interval exceeded one. Additionally, levels of physical activity are an important variable that is not captured within the network and may confound outcomes. We excluded individuals with a history of an outcome of interest to ascertain the effect of early intervention in a relatively less comorbid population than is routinely seen when such drugs are initiated in typical clinical practice. Also, initiation of quadruple therapy in such a short time frame would suggest rapidly progressive disease and a more atypical cohort of T2D, likely to have more pronounced pathophysiology and limits generalisability to a standard population with T2D.

The current strategy of initiating sequential and single-agent therapy risks significant treatment inertia and neglects the critical multiple pathophysiological defects inherent within the ominous octet. In other cardiovascular diseases such as angina pectoris/postmyocardial infarction or heart failure, patients are routinely initiated on multidrug combinations (representing the pharmacological pillars of treatment) from the point of diagnosis [32].

## 5. Conclusion

In summary, we demonstrate beneficial cardiovascular outcomes from combination therapy with metformin, pioglitazone, SGLT2 inhibitor, and GLP-1 RA compared to glucose-centric pharmacotherapy with insulin and SU from the date of diagnosis of T2D. Our study emphasises the importance of remedying the constellation of aberrant metabolic pathways in T2D rather than the primary treatment of hyperglycaemia per se. Future randomised controlled trials are warranted, focusing on high-risk populations and investigating the optimal treatment algorithm and drug sequencing, with a focus on rapid intensification and targeting physiological defects.

## Figures and Tables

**Table 1:** Baseline patient demographics and characteristics.

	**MTF/TZD/GLP-1 RA/SGLT2i (** **n** = 882**)**	**Insulin + sulphonylurea (** **n** = 35,009**)**
Age at index event (years)	58.9 ± 11.2	65.0 ± 13.2
Sex (male/female) (%)	65/35	54/46
Race (White/Black or African American/Asian/Native American/unknown) (%)	73/9/6/2/10	63/17/3/1/16
Weight (kg)	105.2 ± 30.4	94.8 ± 25.1
BMI (kg/m^2^)	32.3 ± 6.7	31.8 ± 7.0
Systolic blood pressure (mmHg)	128.5 ± 16.4	131.0 ± 21.3
Estimated glomerular filtration rate (mL/min/1.73 m^2^)	85.2 ± 25.0	72.0 ± 32.8
LDL cholesterol (mmol/L)	2.1 ± 0.9	2.2 ± 1.0
HbA1c (DCCT %)	8.1 ± 1.5	8.4 ± 2.0
IFCC (mmol/mol)	65 ± 17	68 ± 22

*Note:* Data are expressed as mean ± SD.

*Abbreviations:* BMI, body mass index; LDL, low-density lipoprotein.

**Table 2:** Baseline characteristics before and after propensity score matching.

	**Before PSM**				**After PSM**			
	**MTF/TZD/GLP-1 RA/SGLT2i (** **n** = 882**)**	**Insulin + sulphonylurea (** **n** = 35,009**)**	**p**	**SSMD**	**MTF/TZD/GLP-1 RA/SGLT2i (** **n** = 881**)**	**Insulin + sulphonylurea (** **n** = 881**)**	**p**	**SSMD**
*Demographics*								
Age (years)	58.9 ± 11.2	65.0 ± 13.2	< 0.001	0.493	59.0 ± 11.1	59.1 ± 13.0	0.750	**0.015**
White (%)	73	63	< 0.001	0.200	73	75	0.359	**0.044**
Black or African American (%)	9	17	< 0.001	0.253	9	9	0.801	**0.012**
Sex (male) (%)	65	54	< 0.001	0.256	65	65	0.841	**0.010**
*Comorbidities*								
Essential hypertension (%)	60	60	0.734	**0.012**	60	59	0.884	**0.007**
Atrial fibrillation and flutter (%)	4	12	< 0.001	0.295	4	5	0.312	**0.048**
Heart failure (%)	3	16	< 0.001	0.456	3	3	0.887	**0.007**
Ischaemic heart disease (%)	12	24	< 0.001	0.332	12	13	0.512	**0.031**
Chronic kidney disease (%)	8	20	< 0.001	0.367	8	8	0.857	**0.009**
*Social history*								
Problems related to education and literacy (%)	0	0	0.309	**0.049**	0	0	1	**< 0.001**
Problem related to housing and economic circumstances (%)	1	0	< 0.001	0.112	1	0	0.002	0.152
Nicotine dependence (%)	6	9	0.001	0.123	6	6	0.761	**0.015**
Alcohol dependence (%)	1	1	0.581	**0.018**	1	1	1	**< 0.001**
*Drug treatment*								
Lipid-lowering agents (%)	69	61	< 0.001	0.170	69	66	0.286	**0.051**
ACE inhibitors (%)	31	28	0.046	**0.067**	31	31	0.797	**0.012**
Diuretics (%)	25	37	< 0.001	0.268	25	25	1	**< 0.001**
Beta-blockers (%)	25	41	< 0.001	0.360	25	24	0.868	**0.008**
Angiotensin II inhibitors (%)	27	**25**	0.120	**0.052**	27	26	0.626	**0.023**
Antiarrhythmics (%)	17	30	< 0.001	0.295	17	17	0.899	**0.006**
Platelet aggregation inhibitors (%)	17	32	< 0.001	0.364	17	14	0.129	**0.072**
Anticoagulants (%)	12	36	< 0.001	0.600	12	10	0.246	**0.055**
*Biochemistry*								
Serum LDL cholesterol ≥ 3.4 mmol/L (%)	92	94	0.056	0.062	92	94	0.130	**0.072**
HbA1c ≥ 7.5% (≥58 mmol/mol) (%)	50	59	< 0.001	0.176	50	49	0.634	**0.023**
BMI ≥ 25 kg/m^2^ (%)	66	71	0.002	0.105	66	68	0.387	**0.041**
Systolic blood pressure ≥ 140 mmHg (%)	65	55	< 0.001	0.207	65	65	0.960	**0.002**
Estimated glomerular filtration rate ≤ 60 mL/min/1.73 m^2^ (%)	39	48	0.016	0.172	39	39	0.573	**0.010**

*Note:* Data are expressed as mean ± SD; SSMD in bold indicates variables that are well-matched.

*Abbreviations:* ACE, angiotensin-converting enzyme; BMI, body mass index; LDL, low-density lipoprotein; SSMD, strictly standardised mean difference.

**Table 3:** Change in HbA1c, weight, and eGFR over 3 years, post PSM.

	**MTF/TZD/GLP-1 RA/SGLT2i (** **n** = 881**)**			**Insulin + sulphonylurea (** **n** = 881**)**		
	**Baseline**	**3 years**	**Change**	**Baseline**	**3 years**	**Change**
HbA1c (DCCT %)	8.1 ± 1.5	7.7 ± 1.5	−0.4%	8.8 ± 2.1	8.2 ± 1.9	−0.6%
HbA1c (mmol/mol)	65 ± 17	61 ± 17	−4 mmol/mol	73 ± 23	66 ± 21	−7 mmol/mol
Weight (kg)	104.8 ± 30.9	97.2 ± 26.3	−7.6 kg	97.5 ± 26.9	96.3 ± 25.4	−1.2 kg
Estimated glomerular filtration rate (mL/min/1.73 m^2^)	85.2 ± 25.0	83.1 ± 26.1	−2.1 mL/min/1.73 m^2^	81.0 ± 30.7	79.2 ± 32.7	−1.8 mL/min/1.73 m^2^

## Data Availability

To gain access to the data in the TriNetX research network, a request can be made to TriNetX (https://live.trinetx.com), but costs may be incurred, a data sharing agreement would be necessary, and no patient identifiable information can be obtained.
